# JACIE accreditation for blood and marrow transplantation: past, present and future directions of an international model for healthcare quality improvement

**DOI:** 10.1038/bmt.2017.54

**Published:** 2017-03-27

**Authors:** J A Snowden, E McGrath, R F Duarte, R Saccardi, K Orchard, N Worel, J Kuball, C Chabannon, M Mohty

**Affiliations:** 1 Department of Haematology, Sheffield Teaching Hospitals NHS Foundation Trust, Sheffield, UK; 2 Department of Oncology and Metabolism, University of Sheffield, Sheffield, UK; 3 EBMT Executive Office, Barcelona, Spain; 4 Servicio de Hematologia y Hemoterapia, Hospital Universitario Puerta de Hierro, Madrid, Spain; 5 Department of Hematology, Azienda Ospedaliera Universitaria Careggi, Florence, Italy; 6 Department of Haematology, Southampton General Hospital, Southampton, UK; 7 Klinik fuer Innere Medizin I, Medizinische Universitaet Wien, Vienna, Austria; 8 Department of Haematology, University Medical Centre, Utrecht, The Netherlands; 9 Programme de Transplantation & Therapie Cellulaire, Institut Paoli Calmettes, Marseilles, France; 10 Department of Hematology, Hospital Saint Antoine, Paris, France

## Abstract

Blood and marrow transplantation (BMT) is a complex and evolving medical speciality that makes substantial demands on healthcare resources. To meet a professional responsibility to both patients and public health services, the European Society for Blood and Marrow Transplantation (EBMT) initiated and developed the Joint Accreditation Committee of the International Society for Cellular Therapy and EBMT—better known by the acronym, JACIE. Since its inception, JACIE has performed over 530 voluntary accreditation inspections (62% first time; 38% reaccreditation) in 25 countries, representing 40% of transplant centres in Europe. As well as widespread professional acceptance, JACIE has become incorporated into the regulatory framework for delivery of BMT and other haematopoietic cellular therapies in several countries. In recent years, JACIE has been validated using the EBMT registry as an effective means of quality improvement with a substantial positive impact on survival outcomes. Future directions include development of Europe-wide risk-adjusted outcome benchmarking through the EBMT registry and further extension beyond Europe, including goals to faciliate access for BMT programmes in in low- and middle-income economies (LMIEs) via a ‘first-step’ process.

## Introduction

The impact of quality standards is now felt in many areas of clinical and laboratory practice, along with a substantial bureaucracy on top of clinical work. However, measurable benefits on patient outcomes remain uncertain in many areas.^1^

High-risk procedural specialities (such as cardiac surgery) lend themselves to the introduction of quality measurement and reporting initiatives because serious procedural morbidities and mortality are an intrinsic part of delivering such complex treatments to patients with generally poor prognosis, which can relatively easily be quantified. Potentially, a relatively small specialised clinical community makes it easier to organise agreed quality standards and collect standardised activity and outcome data.

Blood and marrow transplantation (BMT) is a complex multidisciplinary medical speciality with patients commonly being consented for levels of treatment-related mortality risk as high as 40%,^2^ as well as significant long-term post-transplant morbidity, balanced against the adverse prognosis of the many diseases for which BMT is indicated.^3^ Procedure-related risks are highest in the first year, but frequently persist for several years following transplant, reflecting the profound immune suppression and other chronic complications associated with the procedure. Access to high-quality specialist care, within reasonable travelling times, is associated with improved long-term outcomes.^4, 5, 6, 7^

The use of living donors, sourced from both family members and international unrelated bone marrow donor registries, adds another important and almost unique dimension to the speciality, requiring robust systems to ensure quality as well as compliance with legal and regulatory frameworks. Despite such checks and balances, therapeutic activity derived from living cells is susceptible to intrinsic biological variability that can never be completely corrected by manufacturing processes or batch validation in contrast to ‘traditional’ pharmaceutical products.

The aim of this article is to summarise the evolution and current status of JACIE; the Joint Accreditation Committee of ISCT (International Society for Cellular Therapy; www.celltherapysociety.org) and EBMT (European Group for Blood & Marrow Transplantation; www.ebmt.org) in relation to its international acceptance and validation as an effective means of quality improvement with an impact on survival outcomes. Future directions include the development of risk-adjusted outcome benchmarking and the extension of the concept to transplant centres in low- and middle-income economies (LMIEs) via a ‘first-step’ process.

## Quality in BMT: an international model

Almost 20 years ago, European leaders in BMT established JACIE. The goal was an internationally harmonised accreditation system based on agreed quality standards and implemented by teams of voluntary inspectors.^8^ The basic concept was adapted from an earlier initiative in the United States—the Foundation for the Accreditation of Cellular Therapy (FACT; www.factwebsite.org/)—and since then JACIE and FACT have actively collaborated on maintaining a single set of international quality standards (www.jacie.org/standards).

Standards were established in the three main areas of transplant practice as follows: (1) patient care and facilities during the transplant period; (2) donor care during collection of blood and marrow cells; and (3) laboratory processing, storage and delivery of the blood and marrow cells. In each transplant centre, in-house quality management systems underpin the implementation of the standards and integrate the members of clinical and scientific teams involved with the delivery of the service. Accurate data collection is key, including centralised reporting of patient outcome. In some countries, individual centre results of national outcome analyses, such as those from the British Society for Blood and Marrow Transplantation,^9^ are made available to external review.

BMT is a rapidly evolving field, and now involves not only blood and marrow stem cells but many other cellular, immune and cytotoxic therapies, and increasingly overlaps with other areas of ‘regenerative medicine’. The FACT–JACIE standards have moved with the times, with revisions produced every 3 years. Currently, the standards are in their 6th edition, with preparation of the 7th edition underway with publication expected in early 2018. Development of each edition is based on a transparent, structured programme of work, involving international experts and public consultation. The standards are openly available at no cost from the FACT and JACIE web sites. Importantly, flexibility is built in to allow for national regulatory requirements.

## Adoption across Europe

Since its outset, JACIE was set up as a European network coordinated from a central office in Barcelona, Spain. Other than on-site inspection visits, almost all administrative aspects of the programme are delivered remotely using e-mail, internet tools and teleconferencing. JACIE is constituted as a committee of the EBMT with two representatives of the ISCT. Financially, JACIE is run on a not-for-profit basis, resourced almost entirely by application fees. The EBMT contributes administrative support.

Currently, there are ~260 active trained inspectors. The operational language of JACIE is English, although inspections are usually conducted in local languages. Applicant centres are required to translate a selection of their local documents into English only when it is not possible to assign a team familiar with the centre’s working language. Inspection reports are drafted in English by the inspection team, which are reviewed centrally by experienced BMT professionals working with the JACIE Office.

Inspections are overseen by an accreditation committee, and there is a defined pathway for appeal in the event of disagreement. However, this has rarely been invoked, with just one case brought formally to appeal in the last 10 years. The Barcelona office organises training events on a regular basis, including at the EBMT Annual meeting, and provides constructive advice during centre preparation. As a result, only 4 of 647 applications have been rejected, mainly because activity fell below the thresholds established in the standards. Surveys have consistently shown high levels of satisfaction with the process—in 2016, of 46 centres completing a satisfaction survey following the preparation phase and inspection, 78% were very satisfied, 22% were satisfied and 0% dissatisfied.

**Figure 1:**
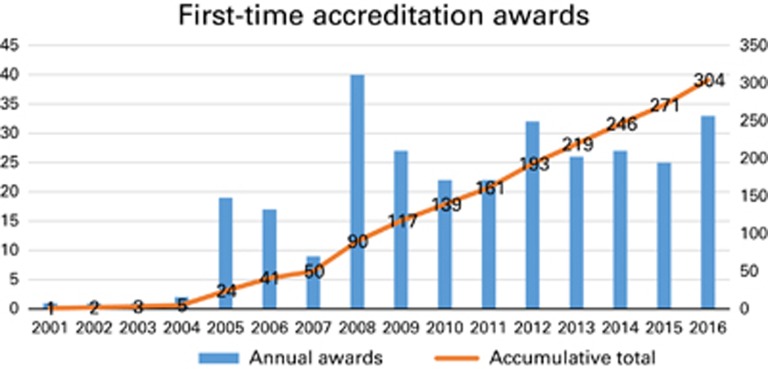
Growth in first-time accredited blood and marrow transplant centres across Europe and elsewhere 2001–2016.

Since its inception, JACIE has performed over 530 accreditation inspections (62% first time; 38% reaccreditation) in 25 countries, representing ~40% of transplant centres in Europe. Although most transplant programmes are in Northern, Southern and Western Europe, centres from Central Europe, the Middle East, Asia and South Africa have also requested accreditation. The process is based on the FACT–JACIE standards and compliance is uniformly checked for all countries, although national regulations will always prevail over standards where a conflict arises. Thus, all transplant programmes are expected to meet the same standards, regardless of location. See Figure 1.

JACIE accreditation is now mandatory in a number of European countries in relation to regulation of BMT practice, public health commissioning or private insurance reimbursement (for example, Belgium, Croatia, France, Italy, Switzerland, The Netherlands and United Kingdom) and national treatment guidelines (www.jacie.org/about/national-regulations). In addition, some clinical trials have included JACIE accreditation among their criteria for entry and this is likely to be increasingly requested for clinical trials of innovative cellular therapies, such as immune effector cells, where high-level standards for product tracking and clinical monitoring after administration are required.

**Figure 2:**
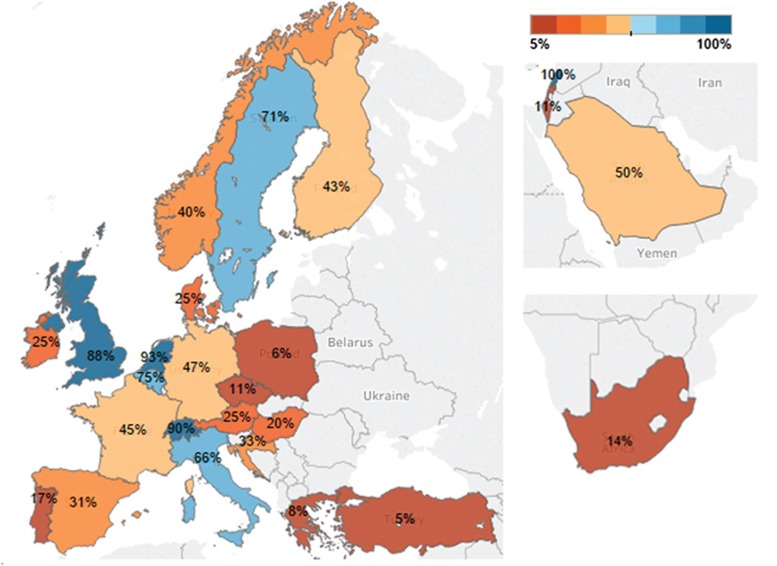
Take-up of accreditation by centres reporting transplants (auto and/or allo) performed in 2014 to the EBMT Activity Survey. Percentage represents number of centres that have applied at least once to JACIE.

In several European countries, including The Netherlands, the United Kingdom and Switzerland, 100% of all BMT centres (Centres reporting data to the EBMT Annual Activity Survey 2014;^10^ www.ebmt.org/Contents/Research/TransplantActivitySurvey/Results/Pages/Results.aspx) performing allogeneic transplantation are accredited by JACIE. Uptake has also been high in Belgium, Sweden and Italy, but relatively low (<45%) in well-resourced countries such as Spain and Austria. Centres in France and Germany are intermediate in their uptake. Further analysis is warranted in this area to identify barriers that impede engagement or other reasons for lack of interest among centres. In recent years, evolving interest has been followed by successful accreditation in Central Europe, Turkey, Saudi Arabia, Lebanon, South Africa and Singapore. See Figure 2.

## Clinical benefits of JACIE quality standards

Has any of this activity made any impact on clinical outcomes?—a crucial question given the effort and resources required to establish and maintain the required standards in each transplant centre. Unusually for the fields of ‘Quality in Healthcare’ and ‘Accreditation Science’, evidence does exist for BMT. Studies using European BMT registry data have correlated roll-out of accreditation with improvements in patient survival and reduction in procedural mortality.^4, 5^ The magnitude of improvement achieved by the relatively simple internal adoption of quality systems, combined with external inspection and accreditation, was as great as many other recent and more highly publicised scientific or pharmacological advances in the field. Evidence relating clinical trials participation and FACT accreditation in the United States also indicated a positive impact, although with mixed findings.^11^ Implementation of the standards and accreditation are also strongly associated with closer alignment with international consensus donor care recommendations for related donors compared with centres without accreditation.^12, 13, 14, 15^ For these reasons, JACIE may be regarded as a prototypical development in clinical medicine, with the potential to be extended to other medical specialties.

An additional advantage of the FACT–JACIE standards is that they bring together clinicians, and personnel from the collection and processing facilities, in pursuance of a common goal; this is rarely seen in the context of authorisation/accreditation by other national or international competent authorities, where the scope is most often restricted to a narrower and more technical field of activity.^16^

## Benefits of international versus national quality standards

Internationally scoped quality systems such as JACIE may offer advantages over locally or nationally delivered schemes. In complex high-risk specialities such as BMT where overall caseload is relatively small and in some countries where centres of practice may be few in number and/or cover large geographic areas, it may be challenging to develop, resource and implement robust national quality systems. The improvement in patient outcomes with JACIE accreditation would have been statistically difficult to prove in any national system, or at least would have taken significantly longer, even in relatively populous countries.

From a practical point of view, BMT clinicians are highly mobile, frequently training and working in more than one country during their career. A common international quality ‘language’ has been effectively transferred across national borders and health services, reducing the effect of individual experiential bias or ‘agenda’ and influence of dominant local financial, clinical or academic conflicts of interest. Moreover, access to a large pool of trained volunteer inspectors across Europe, with independent oversight and defined pathways for appeal via governing bodies in the event of disagreement, can also avoid compromising objectivity through local familiarity.

## Potential disadvantages of JACIE

Many facets of healthcare are already subject to regulation. In particular, the field of transplantation has been the focus of numerous national and European directives, and transposition to national laws has resulted in regular inspection visits from competent authorities. Any new scheme may potentially add a further layer of bureaucracy and little additional clinical value. In the case of JACIE, this impact has been countered by ensuring that the standards are frequently revised and consistent with current trans-national regulations, including European Union Directives. Overlap between regulations and accreditation means that they complement rather than conflict with each other reducing ‘inspection overload’. A facility that complies with the European Union Tissue and Cells Directive for instance will meet most or all of the JACIE standards. In any case, any voluntary international accreditation scheme must cede precedence to local regulatory or legal requirements.

English is the lingua franca of science and medicine, and this is reflected in the JACIE accreditation scheme with practically all standards and guidance documentation being in English. An assumption is made that physicians and senior scientists in most countries have sufficient English to interpret the requirements of the standards. On the other hand, this may not be the case among other relevant groups. For instance, many healthcare professionals, such as nurses, or patients and their families (including donors in the case of allogeneic transplantation) may not have sufficient proficiency in English to comprehend the detail of the standards. To widen understanding of the requirements, translation of the text into other languages, for example, Spanish, French and Turkish has been sporadically undertaken by third parties, for example, national haematology societies and has been accepted by FACT and JACIE with the proviso that the original English will always be the reference text. However, translation carries the risk of altered, incomplete or delayed fidelity to the evolving original English versions and updating translated versions presents such practical challenges and costs that the JACIE Office decided soon after its establishment not to take on this task. In any case, most countries have a National Representative serving as local contact to help address any issues arising from a local interpretation of the standard.

## Benchmarking of survival outcomes

In some other specialities, the use of disease registries enables professionals to engage in continuous learning and share best clinical practices, leading to improved quality care.^17^

The EBMT registry was established in 1974 as a cornerstone of the society and by 2016 had grown to over half a million registered transplants. Evolution of JACIE alongside the registry has enabled a reciprocal relationship to develop. First, JACIE has always required that the inspection team verify that data collected by each applicant centre matches that stipulated by the EBMT registry (that is, Minimal Essential Data forms) or equivalent. Second, the EBMT megafile has been used to validate the benefits of the roll-out of JACIE in terms of survival outcome benefits. Third, the EBMT registry represents a resource for outcome benchmarking.

The EBMT has embarked on developing a benchmarking system for its member centres, based on its two major integral elements: the sizable registry and JACIE accreditation. The North American counterpart of the EBMT, the Center for International Blood and Marrow Transplant Research, has for several years performed centre benchmarking in allogeneic BMT via the Stem Cell Therapeutic Outcomes Database scheme (www.cibmtr.org/About/WhatWeDo/SCTOD/Pages/index.aspx) although in that case, Center for International Blood and Marrow Transplant Research is distinct from FACT.

Although scientifically and statistically challenging, benchmarking has the potential to assure quality of centres performing equivalent treatments. Although presenting a major opportunity for quality improvement, comparing outcomes of clinical practice will inevitably be a sensitive issue. Risk-adaption will be essential given that transplant programmes may be shaped by historical events, staff expertise, referral patterns, case-mix and financial restraints rather than exclusively scientific or outcome-driven factors. Notwithstanding, there is the opportunity for the BMT community to learn from both the high and low achievers, as well as providing information to other stakeholders, such as public health commisioners, healthcare insurers, health technology assessment bodies and other regulators.

## An unmet need: quality systems for BMT in LMIEs

Centres that are currently accredited, inspected or have applied for JACIE accreditation are overwhelmingly in high-income economies in Europe. In addition, those economies usually have other regulatory frameworks within which transplant care is delivered. For many LMIEs, BMT is a prohibitively expensive and complex therapeutic strategy, and sometimes only delivered through private providers, with little or no access to care for the general population. Despite this, transplant activity is increasing outside the high-income economies^10^ in part due to initiatives by the local medical community to adapt established medical practice to their own context including economic constraints.^18^ It is therefore important that quality improvement and accreditation is not inhibitory to the development of BMT in LMIEs.

Through its expanding global network, the EBMT has increasing contact with transplant professionals in LMIEs who have expressed high levels of interest in implementing the FACT–JACIE standards in their units but see the process as organisationally and economically challenging. In response, JACIE has been developing a stepped process based on minimum standards to certify quality-assured BMT services particularly where they are provided to the broader population through public or not-for-profit healthcare providers.

The process is based not on inventing new standards, but instead on a selection of the existing FACT–JACIE standards, particularly those pertaining to quality management, policies and procedures (sections B/C/D 4 and 5 of the standards). In principle, most of these selected standards demand neither major financial investments in technology nor infrastucture, but are more focussed on driving pragmatic changes in working practices and implementing the culture of quality improvement. This model referred to as ‘First-Step’ aims to ensure that centres establish quality management systems for critical processes as a springboard to eventually meeting the more advanced requirements of the standards.

Challenges remain. The feasibility of this approach has yet to be tested on the ground, and inevitably various assumptions will need revisiting after the first inspections against the graded standards. Economic issues will also remain and investment in quality systems may lose out when more immediate medical care needs are pressing. To sustain this approach long-term, there will be a need to recruit inspectors locally along with encouraging engagement by the local medical communities and a need for national leaders to advance the model in the future. At least initially, it is expected that there will be a continued dependence on experienced inspectors from Europe and elsewhere to perform the visits, with inspections in the local languages always where possible, until the local inspector cohort is sufficiently experienced to fully take on the task.

Through this stepped model, the EBMT aims to provide an internationally validated, entry-level certification process with minimised costs. It is hoped that such an initiative, especially if allied to increasing EBMT educational and registry activities, will have a direct impact on patient and donor care, clinical practice and survival outcomes in LMIE centres.

## Conclusions

The potential for quality and external accreditation standards to drive clinical improvement is increasingly described in a number of specialities.^19, 20^ Evidence indicates that success is in part related to ownership by the clinical community.^21^ For BMT, this has been achieved through the EBMT initiating, developing and promoting JACIE across many countries. JACIE accreditation status now ranks with centre activity and experience as key factors related to survival outcomes within any country.^22^

Despite initial concerns that clinical quality standards and accreditation might just be a demanding paper exercise with significant costs and limited benefits,^23, 24^ the relatively unimpeded adoption of JACIE by BMT programmes in countries with widely differing health services, regulations, laws, cultures and languages, with data now available to support improvement in clinical outcomes, is testament to the success of this international approach.

The success of JACIE is an excellent example for clinical quality and accreditation systems in other specialities. A number of potential factors stand out: the early acceptance by some BMT opinion leaders of the need for quality, international professional society support;^25^ the early realisation at local level of the organisational impact^26^ and incorporation into national regulatory requirements. Once acceptance of, and trust in, a system is established, the more controversial aspects, such as standardised performance benchmarking of survival outcomes and minimal centre activity, can be presented as further means of quality improvement.^27, 28, 29^

## Key messages

- Led by front-line BMT professionals for the BMT community.
- Internationally agreed standards supported by the EBMT and other professional organisations across Europe and beyond.
- Complements and traverses national regulatory, legal, cultural and language boundaries. A large pool of international non-remunerated volunteer inspectors overseen by a governance structure avoids local conflicts of interest and makes peer-review more feasible in smaller countries.
- Promotes a positive interaction among different professionals involved in the transplant process, from the laboratory to the bedside.
- Provides standardisation for registry-based analysis of real-world clinical outcome data, enabling benchmarking and future development of complex treatment strategies.
- Potential for adaptation to provide a ‘first-step’ access to centres in low- and middle-income countries, while not compromising original mission for implementing high-quality standards irrespective of economic factors.
